# The Effects of Dietary Thyme Oil (Thymus vulgaris) Essential Oils for Common Carp (Cyprinus carpio): Growth Performance, Digestive Enzyme Activity, Antioxidant Defense, Tissue and Mucus Immune Parameters, and Resistance against Aeromonas hydrophila

**DOI:** 10.1155/2022/7942506

**Published:** 2022-09-14

**Authors:** Hamed Ghafarifarsani, Seyed Hossein Hoseinifar, Atefeh Sheikhlar, Mehdi Raissy, Fatemeh Heidarinezhad Chaharmahali, Worawit Maneepitaksanti, Mehwish Faheem, Hien Van Doan

**Affiliations:** ^1^Department of Fisheries, Faculty of Natural Resources, Urmia University, Urmia, Iran; ^2^Department of Fisheries, Faculty of Fisheries and Environmental Sciences, Gorgan University of Agricultural Sciences and Natural Resources, Gorgan, Iran; ^3^Animal Science Department, Van Hall Larenstein University-Wageningen University and Research, Netherlands; ^4^Department of Aquatic Animal Health, Faculty of Veterinary Medicine, Shahrekord Branch, Islamic Azad University, Shahrekord, Iran; ^5^Faculty of Basic Sciences, Shahrekord Branch, Islamic Azad University, Shahrekord, Iran; ^6^Department of Animal and Aquatic Sciences, Faculty of Agriculture, Chiang Mai University, Chiang Mai, Thailand; ^7^Innovative Agriculture Research Center, Faculty of Agriculture Faculty of Agriculture, Chiang Mai University, Chiang Mai, Thailand; ^8^Department of Zoology, Government College University, Lahore 54000, Pakistan

## Abstract

This study evaluated the effects of dietary supplementation of thyme (Thymus vulgaris) essential oil (TVO) on growth performance, digestive enzymes, biochemical parameters, hematological indices, liver enzymes, and pathogen resistance in common carp (Cyprinus carpio). Triplicate groups of fish (15.36 ± 0.10 g) were fed daily with diets supplemented with TVO at 0, 0.5, 1, and 2 percent for 60 days then challenged with Aeromonas hydrophila. The results determined that supplementation of thyme resulted in significantly higher final body weights (FBW) and lower feed conversion ratios (FCR). Furthermore, no mortality was observed in the thyme-supplemented treatments. Regression analysis showed that fish growth parameters were polynomially related to dietary TVO levels. The optimum dietary TVO level, based upon the varied growth parameters, was 1.344 to 1.436%. Digestive enzymes activity, including amylase and protease, significantly increased in fish fed the supplemented diets. The thyme-supplemented diets also significantly increased the biochemical parameters, including total protein, albumin, and acid phosphatase (ACP), compared to the control group. We also observed significant increases in hematological indices, including red blood cells (RBC), white blood cells (WBC), hematocrit (Hct), and hemoglobin (Hb) in common carp fed diets containing thyme oil (*P* < 0.05). Liver enzymes activity including alanine aminotransferase (ALT), alkaline phosphatase (ALP), and aspartate aminotransferase (AST) was also reduced (*P* < 0.05). Immune parameters, including total protein and total immunoglobulin (total Ig) levels, alternative complement pathway hemolytic (ACH_50_), lysozyme, protease, and ALP in the skin mucus, and lysozyme, total Ig, and ACH_50_ in the intestine were higher (*P* < 0.05) in TVO-supplemented fish. Catalase (CAT), superoxide dismutase (SOD), glutathione reductase (GR), and glutathione peroxidase (GPx) in the liver were also elevated (*P* < 0.05) in TVO administered groups. Lastly, thyme-supplementation resulted in higher survival rates after the A. hydrophila challenge compared to the control (*P* < 0.05). In conclusion, dietary inclusion of thyme oil (1 and 2%) effectively improved fish growth, immune systems, and resistance to A. hydrophila.

## 1. Introduction

The annual production of common carp has been estimated at 4.2 million tons, accounting for 7.7% of the entire aquaculture industry [1]. Intensive carp production is associated with various threats and infectious diseases, which endanger the development of sustainable carp farming [2]. Regrettably, using antibiotics and chemical disinfectants is generally the first approach by fish farmers to control infectious agents. Recent statistics have indicated that global antibiotic consumption in aquaculture is estimated at 10,259 tons in 2017 and predicted to rise to 13,600 tons by 2030 [3].

Numerous side effects from antibiotics and chemicals have raised public health concerns due to residues in the flesh, which create environmental hazards [4, 5]. One alternative and preventive approach to these consequences is the utilization of natural compounds to enhance the immune system through non-specific immune responses [6, 7]. Such natural compounds are categorized into several classes: complex carbohydrates; animal extracts; nutritional factors; lectins; cytokines; and plant extracts [8–11].

Plants and their derivates have been comprehensively proven to improve growth performance and elevate immune responses, thereby generating antimicrobial and anti-stress characteristics [12–16]. Over the past few decades, herbal agents have been extensively investigated and have been reported to have worthwhile potential concerning the therapeutic control over fish pathogens [9, 17–24].

Thyme (*Thymus vulgaris*), an aromatic herb belonging to the family Lamiaceae, contains thymol, carvacrol, cymene, eugenol, and 4-allylphenol and has been proven to enhance growth promotion, antimicrobial, immunostimulation, and antioxidant characteristics [25–31]. As reported, dietary supplementation of this plant influenced the growth and immune parameters in gibel carp (*Carassius auratus gibelio*), common carp (*Cyprinus carpio*), and rainbow trout (*Oncorhynchus mykiss*) [32–34].

According to recent literature, the effects of thyme oil on common carp's immunity and bacterial resistance against *A. hydrophila* are not comprehensively studied. Therefore, this study was designed to investigate the effects of thyme oil on growth, digestive enzymes, biochemical parameters, hematological indices, liver enzymes, mucus and intestinal immunity, and resistance against *A*. *hydrophila* in common carp.

## 2. Materials and Methods

### 2.1. Diet Preparation and Feeding Trial

The chemical composition of the commercial thyme (*Thymus vulgaris*) essential oil (Maleki Commercial Co., Fars, Iran) is presented in Table 1, based on their report. The experimental diets were prepared based on a commercial diet containing 37% crude protein, 6% crude lipid, 6% crude fiber, 8% ash, and 1.25% phosphorus, with 7% moisture (Faradaneh, Shahrekord, Iran). The essential oils (0.5, 1, and 2%) were sprayed on the appropriate weights of the basal diet pellets using a fine mist spray [35, 36]. No essential oil was added to the control diet. The pellets (3.0 ± 0.3 mm) were then coated with 1% bovine gelatin solution (Ramsden *et al*., 2009). Lastly, they were dried at room temperature in darkness, kept in waterproof black zipper storage bags, and stored at 4°C. Diets were fed to fish twice a day for 60 days, with a 3% body weight ratio per day.

### 2.2. Fish and the Rearing System

Three hundred healthy common carp fingerlings were obtained from a local farm in Karaj, Iran, and kept for two weeks to adapt to our experimental conditions. During the acclimatization period, fish were fed the control diet's maintenance ratio (0.5% of the biomass per day). Twenty-five fish with an average body weight of 15.36 ± 0.10 g were randomly allocated to each aquarium (150 liters). Treatments in three replications continued twice a day for 60 days, with a 3% body weight ratio per day [16]. The mean values for water temperature, dissolved oxygen, pH, and hardness were 23.4 ± 1.5°C, 6.4-6.7 mg L^−1^, 7.1-7.3, and 188.56 ± 12.35, respectively. The photoperiod was adjusted to 12L:12D using artificial light. Aeration was performed using an air stone, and 50% of each aquarium's water was replaced daily with fresh de-chlorinated water from reservoir tanks to maintain water quality.

### 2.3. Sampling Procedure

At the end of the 60-day feeding trial, all fish were sampled to evaluate the parameters. Three fish from each tank were then randomly selected and anesthetized using 100 mg L^−1^ eugenol [38] to study the blood parameters. Blood samples were taken from the caudal vein using a 2 ml syringe then centrifuged (Heraeus Labofuge 400) at 1600 g for ten minutes to obtain serum. The serum samples were stored at -20°C for future evaluation.

Cutaneous mucus was collected through the indirect method, according to [39]. Four fish from each tank were anesthetized and individually transferred to plastic bags. They were then mildly rubbed in the bag for approximately one minute then moved back to the tanks. The mucus samples were immediately centrifuged at 1600 g for ten minutes at 4°C in 15 ml sterilized tubes and then stored at -80°C for further evaluation.

For intestine and liver sampling, three fish per tank were euthanized using 100 mg L^−1^ eugenol and head concussion [40, 41]. Samples were then taken out and washed with distilled water. Cold saline solution (0.9%) [10 volumes (w/v)] was used to homogenize the samples on ice using a disperser (IKA T25 digital, Ultra Turrax). Homogenates were centrifuged at 6000 g for 20 minutes at 4°C. The resulting supernatants were stored at -80°C for future analysis.

### 2.4. Growth Parameters

A digital scale (Sartorius Digital Gram Scale TE1502S) with 0.1 g precision was used to measure fish weight. Growth parameters, weight gain, specific growth rate, feed conversion ratio, and survival rate were then calculated through the following equations; and mortalities (if any) were recorded throughout the trial to compare the treatment survival rates.

(1) $$\begin{array}{c} \text{Weight gain }\left(\text{WG}\right)\;\left(\text{g}\right)=\text{Initial body weight}–\text{Final body weight}\\ \text{Specific growth rate }\left(\text{SGR}\right)\;\left(\%\text{d}-1\right)=\left[\left\{\ln\text{ final weight }\left(\text{g}\right)–\ln\text{ initial weight }\left(\text{g}\right)\right\}/\text{days}\right]\times 100\\ \text{Feed conversion rate }\left(\text{FCR}\right)=\text{Total feed given }\left(\text{g}\right)/\text{Weight gain }\left(\text{g}\right)\\ \text{Survival rate }\left(\text{SR}\right)\;\left(\%\right)=\left(\text{final numbers}/\text{initial numbers}\right)\times 100 \end{array}$$

### 2.5. Digestive Enzymes

Digestive tract samples were homogenized in 25 mM Tris-HCl buffer at pH 7.2 and centrifuged at 25,000 g for 20 minutes. The resulting supernatants were then collected, and lipase, protease, and amylase activities were determined [16].

### 2.6. Liver-Specific Enzymes

Liver-specific enzymes activity, consisting of alkaline phosphatase (ALP), alanine aminotransferase (ALT), and aspartate aminotransferase (AST), was assessed with a commercial kit (Pars Azmun, Co., Tehran, Iran), based on the method designed by [42].

### 2.7. Hematological Parameters

The measurements of hematological indices were carried out according to the method described [43]. Red and white blood cell counts were performed using a hemocytometer slide. Hematocrit (Hct) was measured based on the microhematocrit technique and expressed as the percentage of packed cell volume. Hemoglobin (Hb) was quantified according to the cyanomethemoglobin method via a commercial kit. The following equations were used to calculate mean corpuscular volume (MCV), mean corpuscular hemoglobin (MCH), and mean corpuscular hemoglobin concentration (MCHC):

(2) $$\begin{array}{c} \text{MCHC}=\text{Hb}\times 10/\text{Hct};\text{MCV}=\text{Hct}\times 10/\text{RBC }\left(\text{million}\right);\text{MCH}=\text{Hb}\times 10/\text{RBC }\left(\text{million}\right). \end{array}$$

### 2.8. Serum Biochemical Parameters

Total protein and albumin levels were measured by a commercial spectrophotometry kit (Pars Azmun Co, Tehran, Iran) based on the colorimetric method [44]. We determined acid phosphatase (ACP) activity according to the protocol of [45], expressed as nanomoles (nM) of p-nitrophenol released/min/mg protein at 25°C with an acetate buffer (0.2 M, pH 5).

### 2.9. Immunological Parameters

Immunological parameters were analyzed in intestine and mucus samples through conventional techniques. According to a slightly modified method of [46]), lysozyme activity was determined. In brief, 0.2 mg/ml of the bacterium (*Micrococcus luteus*) suspension was prepared with the sodium phosphate buffer (0.05 M, pH 6.2). Sixty *μ*L of the sample was mixed with the bacterium suspension (2 ml) and incubated for three minutes. The absorbance was then read, in which a single unit of lysozyme was considered a decrease (0.001 per min) in absorbance. Alternative complement pathway hemolytic activity (ACH_50_) was measured in the intestine and mucus samples through the method developed by Ortuno et al. (2000), which is based on sheep red blood cells (SRBC) hemolysis. For the measurement of total immunoglobulin (total Ig), the samples were sedimented with 12.5% polyethene glycol solution (Sigma). Total Ig was then determined after calculating protein concentrations before and after sedimentation [48].

Protease activity in the mucus was measured via the azocasein hydrolysis approach [39], and mucus alkaline phosphatase (ALP) activity and total protein (TP) levels were determined through the use of a commercial kit (Pars Azmun Co., Tehran, Iran).

### 2.10. Liver Antioxidant Parameters

The function of glutathione reductase (GR), superoxide dismutase (SOD), and catalase (CAT) activity, as well as the function of glutathione peroxidase (GPx) (where glutathione is converted to glutathione disulfide), was measured through the use of commercial equipment (Zellbio®, Berlin, Germany). Malondialdehyde (MDA) concentrations were also measured using thiobarbituric acid, based on the calorimetric method defined by Buege and Aust [49].

### 2.11. Bacterial Infection

*Aeromonas hydrophila* (AH04) was obtained from the Department of Aquatic Animal Health, Faculty of Veterinary Medicine, University of Tehran, Iran. The bacteria were cultured on a tryptone soya agar (TSA) medium with a 0.85% saline solution. After the 60-day feeding trial, fifteen fish from each replicate were injected intraperitoneally with the bacterial suspension (0.1 ml, 10^8^ CFU per fish). The mortality rates were recorded for 14 days [16]. The relative percentage survival (RPS) and survival rate (%) of infected fish were calculated based on the following formulas [17]:

(3) $$\begin{array}{c} \text{RPS }\left(\%\right)=\left(1-\%\text{of mortality in treated groups}∕\%\text{of mortality in control group}\right)\times 100. \end{array}$$

### 2.12. Statistical Analysis

Growth responses to dietary TVO were assessed through polynomial regression. The research herein was conducted in a completely randomized approach. Normality tests were conducted (Kolmogorov-Smirnov), and the analysis of variance (one-way ANOVA) was applied. The Kaplan-Meier method was applied for mortality rate data. Duncan's post hoc test with a 95% level of confidence was performed, and SPSS 20 software was employed for statistical analysis. Data were represented as the mean of three measurements ± standard error (SE).

## 3. Results

### 3.1. Growth Performance

**Figure 1:**
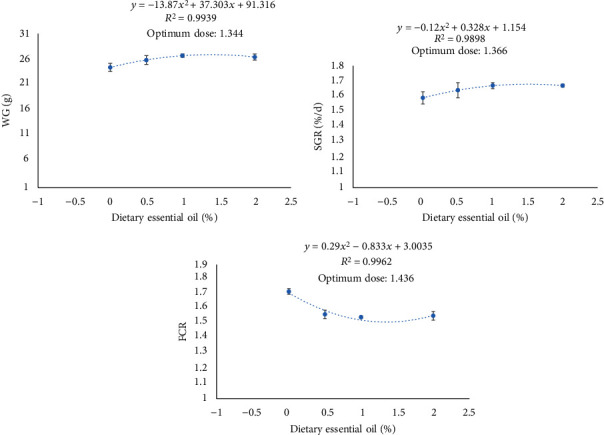
Relationships between the dietary TVO levels and growth parameters of common carp.

The inclusion of thyme oil influenced final body weight (FBW), feed conversion ratio (FCR), and survival rates (SR) (Table 2). The final weights of fish supplemented with the diet containing 1% TVO were significantly higher than that of the control group (*P* < 0.05). FCR was also significantly lower in the fish fed the thyme-supplemented diets (*P* < 0.05). The survival rates (SR) in all groups provided the TVO diets were 100% versus the control group at 93%. Fish responded exponentially to graded levels of dietary TVO levels (Figure 1). Accordingly, the lowest FCR and highest SGR and WG were observed at 1.436, 1.366, and 1.344% dietary TVO levels, respectively.

### 3.2. Digestive Enzymes

Table 3 presents the levels of digestive enzymes, including amylase, lipase, and protease in the experimental fish. Resultingly, amylase and protease were significantly higher in the fish fed the supplemented diets (*P* < 0.05), whereas lipase levels presented no significant differences among each treatment (*P* < 0.05).

### 3.3. Biochemical and Hematological Indices

Tables 4 and 5 display the biochemical and hematological indices. Total protein, albumin, and ACP levels were significantly enhanced compared to those of the control group (*P* < 0.05). Differences in globulin, MCHC, MCH, and MCV values among treatments were not statistically significant. However, significant increases were found within the hematological indices, including RBC, WBC, Hct, and Hb in common carp fed the TVO diets (*P* < 0.05).

### 3.4. Liver-Specific Enzymes

Liver enzyme levels are presented in Table 6. ALT decreased in the fish that received 1 and 2% TVO, though not significantly. AST levels were significantly reduced in the fish supplemented with thyme oil in comparison to that of the control (*P* < 0.05).

### 3.5. Skin Mucus and Intestine Immune Parameters

**Figure 2:**
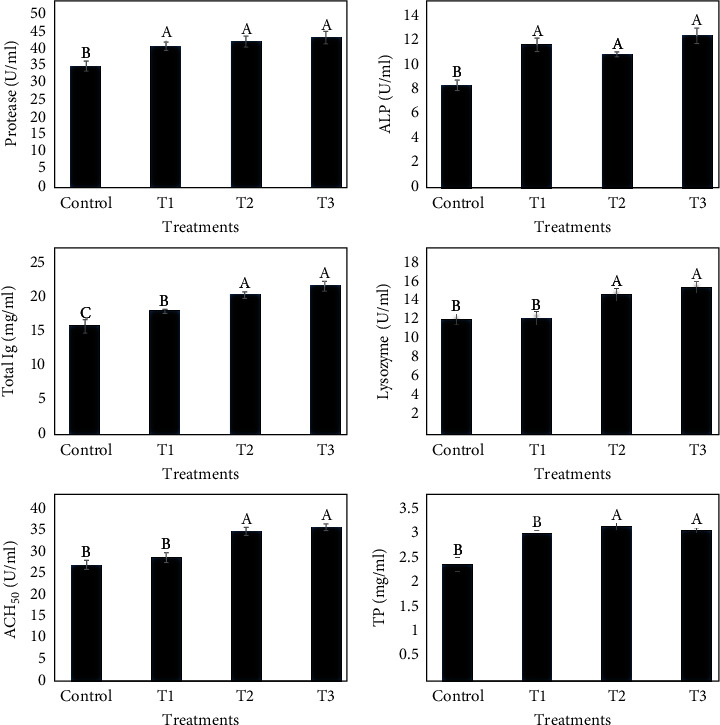
The effects of dietary TVO on skin mucus protease, alkaline phosphatase (ALP), lysozyme, and ACH_50_ activities and total immunoglobulin (total Ig) and total protein (TP) levels in common carp after 60 days. Bars assigned with different superscripts are significantly different (*P* < 0.05). Values are presented as the mean ± SE.

**Figure 3:**
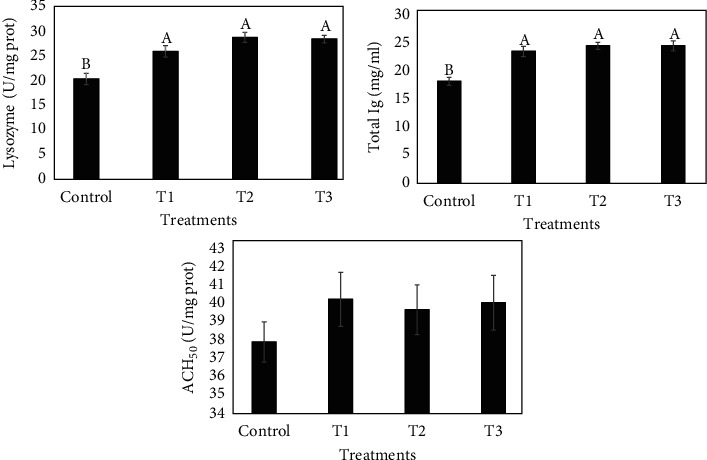
The effects of dietary TVO on intestine lysozyme and ACH_50_ activities and total Ig levels in common carp after 60 days. Bars assigned with different superscripts are significantly different (*P* < 0.05). Values are presented as the mean ± SE.

Skin mucus and intestinal immune parameters are outlined in Figures 2 and 3, respectively. Total Ig and total protein level, ACH_50_, protease, ALP, and lysozyme activity in common carp skin mucus were significantly higher in treatments supplemented with thyme oil than in those of the control group (*P* < 0.05). Lysozyme activity and total Ig level were significantly elevated in the fish supplemented with TVO (*P* < 0.05). Increases in ACH_50_ activity were not statistically significant compared to the control.

### 3.6. Liver Antioxidant Parameters

**Figure 4:**
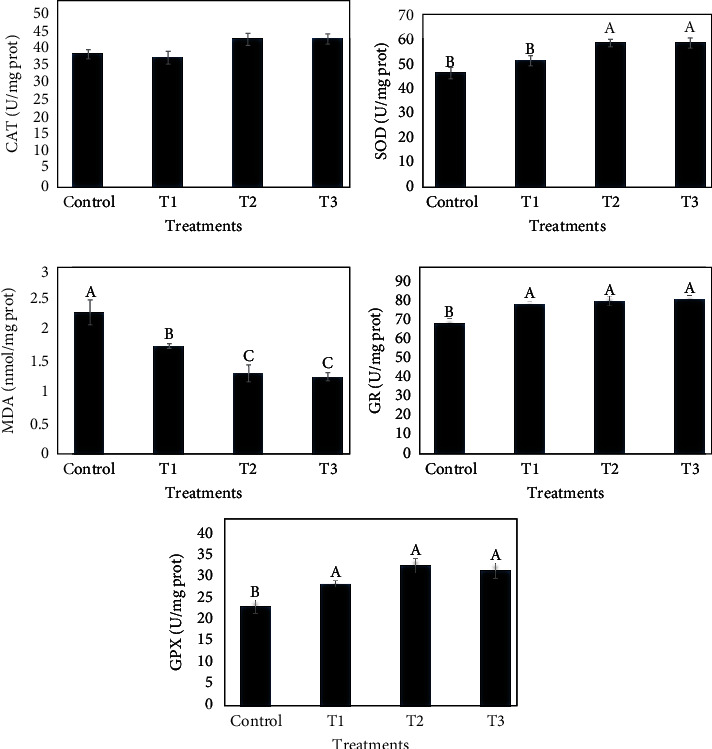
The effects of dietary TVO on liver catalase (CAT), superoxide dismutase (SOD), glutathione peroxide (GPx), glutathione reductase (GR) activities, and malondialdehyde (MDA) content in common carp after 60 days. Bars assigned with different superscripts are significantly different (*P* < 0.05). Values are presented as the mean ± SE.

The level of antioxidant enzymes, including CAT, SOD, MDA, GR, and GPx, are shown in Figure 4. While CAT, SOD, GR, and GPx increased in fish that received the TVO-supplemented diets, MDA content significantly decreased compared to control (*P* < 0.05).

### 3.7. Challenging Test

**Figure 5:**
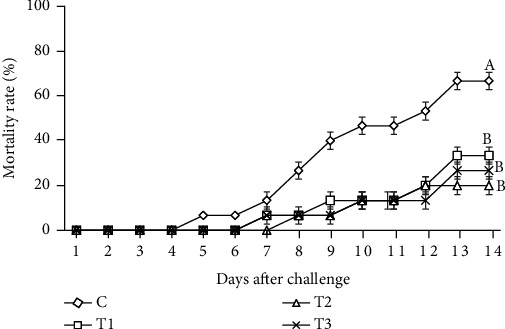
Mortality rates (%) of common carp *Cyprinus carpio* over 14 days after challenge with *A. hydrophila* and fed different experimental diets compared to the control group. Values are presented as the mean ± SE.

According to Table 7 and Figure 5, mortality in the control group started earlier (the five^th^ day) than the other treatments. In contrast, mortality in the specimens fed with T2 diet occurred later (the eight day) than others. At the end of the experimental infection, the CMR of fish fed with diets containing T2 (20%) decreased to about 3.30 folded compared to the control group (% 66.33) (*P* < 0.05). Furthermore, the highest SR (%) and RSP (%) were obtained in fish fed with diet supplemented with T2 (Table 7). However, SR among groups fed thyme oil were statistically similar.

## 4. Discussion

Thyme and its derivatives have frequently been used as supplements in fish diets primarily to enhance growth and immunity [26, 50–58]. In the present study, the dietary supplementation of TVO improved fish survival rates, FCR, and final body weight (FBW), in which the FCR values were lower, and the FBWs higher in fish fed the TVO-supplemented diets (1%).

In agreement with our results, several studies have reported the growth-enhancing effects of TVO supplementation. In rainbow trout, fish fed a diet containing 0.5 mg/kg thyme essential oil exhibited higher WG (30.9%) and SGR (18.36%) compared to the control [34]. Similar results were also observed in the study by Sönmez *et al*. [59], where higher WG was obtained in rainbow trout supplemented with thyme oil. The beneficial effects of thyme and its derivatives have also been reported for Nile tilapia (*Oreochromis niloticus*) [31], *Oncorhynchus mykiss* [59], *Dicentrarchus labrax* (Volpatti *et al*., 2013), and *Cyprinus carpio* AlSafah and Al-Faragi [32]. In our study, improved growth in the TVO-supplemented fish can be attributed to the enhancing effects of TVO on the digestive abilities of the fish. We observed a significant increase in amylase and protease enzyme activities in TVO-supplemented fish compared to the control. Based on recent literature review, very little data is available on the effects of thyme and its derivatives on fish digestive enzymes. In the study of AbdEl-Naby *et al*. [61], a combination of chitosan nanoparticles and thymol significantly enhanced lipase and protease activities, and subsequent feed utilization. The inclusion of 4% thyme in the common carp diet modified digestive enzymes' activity in fish exposed to aflatoxin-B_1_ [62]. Khalil *et al*. [54] observed significant elevations in amylase activity of Nile tilapia, having consumed supplemental thyme (*Thymus vulgaris*) powder. Plant extracts, essential oils, and the bio-compounds have been extensively reported as growth enhancers in fish [63–69]

In the present study, the biochemical components of blood, total protein, albumin, and ACP, which are indicators of the health status of fish, increased in fish fed the diets containing TVO. Serum total protein is an important indicator of fish nutrition and health status [70–72]. Albumin is synthesized in the liver and acts as an antioxidant against oxidative stress induced by free radicals [73], and acts as a carrier in transferring hormones, vitamins, and minerals in the blood [74–76]. Another serum protein, globulin, is considered an essential component of the innate immune system of fish [77–79].

Similar to the biochemical components of blood, hematology indices are also evaluated to determine the health status of fish [80, 81]. In this study, RBC, WBC, Hct, and Hb values increased in TVO-supplemented fish compared to the control group. Hematological alterations have also been reported in fish supplemented with thyme and thyme derivatives. In the study of Valladão *et al*. [82], the numbers of lymphocytes and leukocytes increased in the blood of Nile tilapia fed thyme essential oil supplements, which may be attributed to the immunostimulatory effects of the essential oil on the cellular components of the non-specific immune system. Similarly, Zadmajid and Mohammadi [33], confirmed the immunostimulatory effects of thyme essential oil in gibel carp (*Carassius auratus gibelio*), where the dietary essential oil enhanced RBC and WBC, Hct, and Hb. Bioactive phytochemicals such as polyphenols, with interesting properties, like anti-inflammation [83], may also play immunostimulatory roles in the body.

Based on recent literature, elevated levels of hepatic metabolic enzymes (HMEs) can indicate hepatic damage [84]. In the present study, TVO supplementation had seemingly no deteriorating effect on hepatocytes, demonstrated by the HME blood levels, in which the TVO-supplemented fish showed no differences from that of the control. In agreement with our results, decreased ALT and AST levels in gibel carp (*Carassius auratus gibelio*) were attributed to the protective effects of thyme essential oil on hepatocytes [33]. Zargar *et al*. [34] observed that the addition of thymol oil to the diets of rainbow significantly reduced serum ALP and AST levels. Similarly, the addition of 10 g/kg thyme extract to the diets of rainbow trout significantly decreased AST and ALT activity levels [52]. The supplementation of *Channa argus* with 300 and 450 mg thymol/kg significantly decreased the serum activity of ALT and AST [85]. In African catfish (*Clarias garipenus*), dietary *T. vulgaris* essential oil reduced ALT, AST, and ALP levels in thiamethoxam intoxicated fish [86]. And, in Nile tilapia, ALT and AST activities were significantly reduced by adding thyme powder (5 g kg^−1^ diet) to their diets [87]. Some compounds, such as flavonoids and phenols, are responsible for the protective effects of essential oils on hepatocytes due to their stabilizing effects on the fish cell membrane [33]. Additionally, thyme essential oil may exert antioxidative effects by enhancing the bioavailability of the antioxidants in vitamins C and E [33].

In the present study, TVO supplementation enhanced the immune responses and antioxidant enzyme activities in fish skin and intestines, thereby demonstrating the role of TVO as an immunostimulant. The immunostimulatory effects of thyme have also been reported in previous studies. Zargar *et al*. [34] reported a significant elevation in complement and lysozyme gene expressions following the administration of 1-2 ml/kg of thyme essential oil in rainbow trout. Mirghaed *et al*. [55] observed significant increases in the humoral and skin mucus immune components and immune-related gene expressions in rainbow trout supplemented with 2-3 g/kg thyme (*Zataria multiflora*) extract. Similar results were reported by Hoseini and Yousefi [52] in which thyme (*Thymus vulgaris*) extract significantly increased serum total protein, total Ig, lysozyme, and ACH_50_ activity. In the study of Emeish and El-Deen [50], GPx and CAT significantly increased in fish received thyme. Serum and hepatic SOD and CAT activities were also elevated in rainbow trout in response to diets containing 2-3 g/kg thyme [55]. The immune stimulant property of TVO could be attributed to the presence of the bioactive ingredients, which are proved to boost immune response in the body by reduction of inflammation as well as preventing oxidation process [88].

In the present study, TVO supplementation significantly increased fish survival rate after the *A. hydrophila* challenge. The most significant influence of thyme oil on post-challenge survival rates was evidenced in the 1% TVO-supplemented diet, which may be attributed to the enhancing effects of the essential oil on the fish immune system. Several compounds with antibacterial properties in thyme's chemical composition, such as 1, 8-cineole, borneol, alpha-pinene, beta-pinene, and terpinen-4-ol, may also be responsible for added resistance to bacterial infections [89, 90]. Therefore, the main reason for the antibacterial activity of TVO can be related to the presence of phenolic compounds (7.3 mg/g GAE) as the main bioactive ingredient in this oil. This compound possesses high antibacterial potentials due to the ability to (1) inhibit bacterial virulence factors such as enzymes and toxins, (2) interact with cytoplasmic membrane, (3) suppress biofilm formation, and (4) exert a synergistic effect with antibiotics [91, 92]. The review study of Alagawany *et al*. [18] concluded that the protective effects of thymol (as a main active compound in the composition of thyme) against oxidative stress and infections might be due to its antimicrobial, antiviral, antioxidant, immunomodulatory, and anti-inflammatory properties. The antibacterial effects of thyme and other plant-based supplements in controlling fish infections have been widely investigated [93, 94]. The supplementation of rainbow trout diets with thyme essential oil (*Thymus vulgaris*) significantly decreased fish mortality after a challenge with *Yersinia ruckeri* [27]. Zargar *et al*. [34] reported higher survival rates in rainbow trout (*Oncorhynchus mykiss*) that received thyme essential oil in the diet.

In conclusion, our results successfully show that dietary thyme essential oil (1% and 2%) positively influenced the growth parameters, digestive enzymes, hematology, immunity, and resistance to *A. hydrophila* infection in common carp. Most notably, the highest fish survival rate was achieved by supplementation 1% TVO. Accordingly, both 1 and 2% TVO inclusions may be recommended as dietary supplementations in the diets of common carp (*Cyprinus carpio*).

## Figures and Tables

**Table 1:** Chemical composition of *Thymus vulgaris* essential oil.

Compound	%
Thymol	45
Carvacrol	3.5
ƿ-Cymene	20
*γ*-Terpinene	8
Linalol	4
*β*-Myrcene	1.5
*α*-Terpinene	1.3

**Table 2:** Growth performance of common carp fed four experimental diets over 60 days. Values are presented as the mean ± SE.

Parameters	Control	T1	T3	T2
IW (g)	15.24 ± 0.18	15.40 ± 0.24	15.34 ± 0.24	15.48 ± 0.28
FW (g)	39.73 ± 0.62^b^	41.31 ± 0.66^ab^	41.84 ± 0.82^ab^	42.26 ± 0.44^a^
WG (g)	24.49 ± 0.80	25.91 ± 0.87	26.50 ± 0.60	26.78 ± 0.33
SGR (% d^−1^)	1.59 ± 0.04	1.64 ± 0.05	1.67 ± 0.01	1.67 ± 0.02
FCR	1.75 ± 0.02^a^	1.59 ± 0.03^b^	1.58 ± 0.03^b^	1.57 ± 0.01^b^
SR (%)	93.00 ± 0.57^b^	100.00 ± 0.00^a^	100.00 ± 0.00^a^	100.00 ± 0.00^a^

IW: initial weight (g); FW: final weight (g); WG: weight gain; SGR: specific growth rate; FCR: feed conversion ratio; SR: survival rate. Different letters (a–b) in the same row indicate significant differences (*P* < 0.05).

**Table 3:** Digestive enzymes activity of common carp fed four experimental diets over 60 days. Values are presented as the mean ± SE.

Parameters	Control	T1	T2	T3
Amylase (U/ml)	2.33 ± 0.10^b^	2.58 ± 0.08^ab^	2.66 ± 0.07^a^	2.71 ± 0.09^a^
Lipase (U/ml)	1.70 ± 0.08	1.57 ± 0.07	1.75 ± 0.04	1.82 ± 0.07
Protease (U/ml)	17.56 ± 0.61^b^	20.27 ± 0.53^a^	21.56 ± 0.90^a^	21.74 ± 0.74^a^

Different letters (a–b) in the same row indicate significant differences (*P* < 0.05).

**Table 4:** Biochemical parameters of common carp fed four experimental diets over 60 days. Values are presented as the mean ± SE.

Parameters	Control	T1	T2	T3
Total protein (g/dL)	2.15 ± 0.12^b^	2.55 ± 0.07^a^	2.70 ± 0.07^a^	2.67 ± 0.11^a^
Albumin (g/dL)	1.25 ± 0.04^b^	1.68 ± 0.05^a^	1.86 ± 0.06^a^	1.82 ± 0.04^a^
Globulin (g/dL)	0.89 ± 0.12	0.86 ± 0.04	0.84 ± 0.11	0.85 ± 0.13
ACP (g/L)	10.99 ± 0.44^b^	13.70 ± 0.30^a^	14.69 ± 0.36^a^	14.10 ± 0.48^a^

ACP: acid phosphatase. Different letters (a–b) in the same row indicate significant differences (*P* < 0.05).

**Table 5:** Hematological indices of common carp fed four experimental diets over 60 days. Values are presented as the mean ± SE.

Parameters	Control	T1	T2	T3
RBC (× 10^6^/*μ*l)	1.27 ± 0.04^b^	1.44 ± 0.02^a^	1.42 ± 0.03^a^	1.47 ± 0.04^a^
WBC (× 10^3^/*μ*l)	4.12 ± 0.05^b^	4.44 ± 0.03^a^	4.46 ± 0.03^a^	4.48 ± 0.03^a^
Hct (%)	26.00 ± 0.66^b^	31.00 ± 1.15^a^	31.66 ± 1.20^a^	32.00 ± 1.15^a^
Hb (g/dl)	17.07 ± 0.43^b^	19.66 ± 0.45^a^	19.57 ± 0.51^a^	19.79 ± 0.71^a^
MCHC (g/dl)	65.79 ± 3.13	63.49 ± 0.88	61.86 ± 0.98	61.97 ± 2.77
MCH (pg/cell)	134.25 ± 4.42	136.71 ± 5.01	137.50 ± 6.90	134.83 ± 5.95
MCV (nm^3^)	204.72 ± 9.22	215.60 ± 10.63	222.60 ± 13.68	217.60 ± 1.66

Different letters (a–b) in the same row indicate significant differences (*P* < 0.05).

**Table 6:** Liver-specific enzymes activity of common carp were fed four experimental diets over 60 days. Values are presented as the mean ± SE.

Parameters	Control	T1	T2	T3
ALT (U/ml)	17.06 ± 0.41^ab^	17.66 ± 0.48^a^	15.43 ± 0.52^b^	15.89 ± 0.52^b^
AST (U/ml)	26.78 ± 0.68^a^	23.55 ± 0.70^b^	22.27 ± 0.67^b^	21.34 ± 0.57^b^
ALP (U/ml)	14.21 ± 0.57	14.61 ± 0.36	13.62 ± 0.36	13.91 ± 0.25

Different letters (a–b) in the same row indicate significant differences (*P* < 0.05).

**Table 7:** Survival rate (SR) and relative percentage survival (RPS) of common carp were fed four experimental diets over 60 days and infected with *Aeromonas hydrophila* for14 days. Values are presented as the mean ± SE.

	Control	T1	T2	T3
SR (%)	33.77 ± 3.75^b^	66.66 ± 3.75^a^	80.0 ± 4.04^a^	73.34 ± 3.75^a^
RPS (%)	—	49.75 ± 0.05	69.85 ± 0.06	59.80 ± 0.05

Different letters (a–b) in the same row indicate significant differences (*P* < 0.05).

## Data Availability

The data that support the findings of this study are available upon reasonable request to the corresponding authors.
